# Efficacy of atomoxetine in adult attention-Deficit/Hyperactivity Disorder: a drug-placebo response curve analysis

**DOI:** 10.1186/1744-9081-1-16

**Published:** 2005-10-03

**Authors:** Stephen V Faraone, Joseph Biederman, Thomas Spencer, David Michelson, Lenard Adler, Fred Reimherr, Stephen J Glatt

**Affiliations:** 1 Department of Psychiatry, SUNY Upstate Medical University, Syracuse, NY 13210, USA; 2 Department of Psychiatry, Harvard Medical School, Massachusetts General Hospital, Boston, MA 01880, USA; 3 Lilly Research Laboratories, Indianapolis, IN 46285, USA; 4 New York University School of Medicine, New York, NY 10016, USA; 5 Mood Disorders Clinic, Department of Psychiatry, University of Utah Health Sciences Center, Salt Lake City, UT 84132, USA; 6 Institute of Behavioral Genomics, Department of Psychiatry, University of California, San Diego, La Jolla, CA 92093-0603, USA

## Abstract

**Background:**

The objective of this study was to evaluate the efficacy of atomoxetine, a new and highly selective inhibitor of the norepinephrine transporter, in reducing symptoms of attention-deficit/hyperactivity disorder (ADHD) among adults by using drug-placebo response curve methods.

**Methods:**

We analyzed data from two double-blind, placebo-controlled, parallel design studies of adult patients (Study I, *N *= 280; Study II, *N *= 256) with DSM-IV-defined ADHD who were recruited by referral and advertising. Subjects were randomized to 10 weeks of treatment with atomoxetine or placebo, and were assessed with the Conners Adult ADHD Rating Scales and the Clinical Global Impression of ADHD Severity scale before and after treatment.

**Results:**

Those treated with atomoxetine were more likely to show a reduction in ADHD symptoms than those receiving placebo. Across all measures, the likelihood that an atomoxetine-treated subject improved to a greater extent than a placebo-treated subject was approximately 0.60. Furthermore, atomoxetine prevented worsening of most symptom classes.

**Conclusion:**

From these findings, we conclude that atomoxetine is an effective treatment for ADHD among adults when evaluated using several criteria.

## Introduction

Several compounds are now recognized as effective treatments for the major symptoms of attention-deficit/hyperactivity disorder (ADHD) in adulthood. The most effective of these include methylphenidate and dextroamphetamine (or mixed dextro- and levoamphetamine); however, the use of other agents, such as bupropion and desipramine, has also received some support. In addition to these, atomoxetine, a highly selective noradrenergic reuptake inhibitor with little affinity for other neurotransmitter systems [1], has been shown to be well tolerated and effective in reducing the symptoms of ADHD in adulthood. In fact, the benefits of atomoxetine for adults with ADHD have now been demonstrated in three studies of adult patients [2,3], with each report establishing the superiority of atomoxetine over placebo in reducing inattentive, hyperactive, and impulsive symptoms of the illness [3]. As a result of its demonstrated efficacy and low occurrence of clinically meaningful side effects [4], atomoxetine recently became the first non-stimulant medication approved for use in the United States for the treatment of ADHD in adults.

Thus, the ability of atomoxetine to reduce symptoms of ADHD among adults has been sufficiently established; however, several key questions about its clinical utility remain unresolved. For example, although the initial studies of the efficacy of atomoxetine provided useful information for clinicians treating adults with ADHD, such as the average magnitude of the decrease in ADHD symptoms associated with drug treatment and the reliability of this effect, the standard methods of data presentation in these reports do not provide information about the full range of effects of this compound. To further characterize the clinical performance of atomoxetine, we completed a drug-placebo response curve analysis of the data initially reported by Michelson *et al*. [3] This method, described by Faraone *et al*. [5], is a generalization of receiver operating characteristic (ROC) analysis [6], which has been widely applied to assessing the accuracy of diagnostic tests [7-9]. The goal of this method is to identify additional characteristics of drug-placebo differences that have already been shown to be statistically significant, including: 1) the size of the effect using different response criteria; 2) the nature of individual responses; and 3) the portion of the drug's effect that is due to symptom improvement, the prevention of symptom worsening, or both.

## Results

### Standard Analyses

To provide a foundation for interpreting the results of drug-placebo response curve analysis of the effects of atomoxetine, we have displayed in Table 1 a summary of results of the standard analyses of these data, which were originally presented by Michelson *et al*. [3]. Although data on clinical global impression (CGI) endpoints or Conners Adult ADHD Rating Scale (CAARS) ADHD index scores were not presented in the initial report, it is clear that atomoxetine had significant efficacy relative to placebo on all other measures derived from the CAARS or CGI assessments. The most reliable reductions in ADHD symptoms elicited by atomoxetine were seen for clinician-rated global impressions of ADHD severity, and investigator- and self-rated total ADHD symptoms. Atomoxetine more robustly reduced inattentiveness than hyperactivity and impulsivity, as assessed by both investigators and subjects. Overall, these data provided strong and conclusive evidence that atomoxetine was superior to placebo in reducing the symptoms of ADHD in adulthood, warranting further analysis by drug-placebo response curve methods.

**Table 1:** Summary of Effects of Atomoxetine and Placebo on ADHD Symptoms in Adults*

	Study I			Study II		
	
	Placebo (*n *= 134)	Atomoxetine (*n *= 133)	*p*	Placebo (*n *= 124)	Atomoxetine (*n *= 124)	*p*
	
CGI Change Score	-0.4 ± 1.0	-0.8 ± 1.2	0.010	-0.5 ± 1.0	-0.9 ± 1.2	0.002
*Investigator-Rated CAARS*						
Total ADHD Symptom Scores	-6.0 ± 9.3	-9.5 ± 10.1	0.005	-6.7 ± 9.3	-10.5 ± 10.9	0.002
Inattentive Score	-3.1 ± 5.8	-5.0 ± 5.7	0.010	-3.5 ± 5.3	-5.8 ± 6.5	0.001
Hyperactive/Impulsive Score	-2.9 ± 4.9	-4.5 ± 5.1	0.017	-3.2 ± 4.7	-4.7 ± 5.3	0.013
*Self-Rated CAARS*						
Total ADHD Symptom Score	-9.3 ± 14.0	-16.0 ± 16.2	0.002	-11.6 ± 16.1	-17.3 ± 17.6	0.008
Inattentive Score	-8.6 ± 13.8	-15.9 ± 16.3	0.001	-11.3 ± 16.6	-17.1 ± 17.9	0.012
Hyperactive/Impulsive Score	-7.5 ± 12.1	-11.9 ± 13.5	0.013	-8.8 ± 13.4	-12.5 ± 14.1	0.025

*Data adapted from Michelson *et al*., 2003

Data are presented as mean ± standard deviation

### CAARS Investigator Ratings

**Figure 1:**
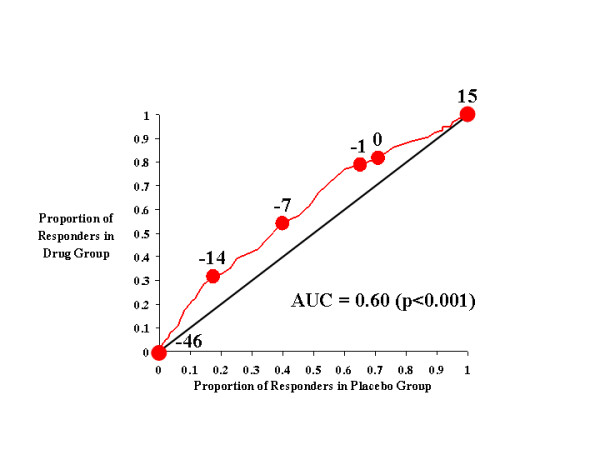
CAARS Investigator-Rated Total ADHD Score.

**Figure 2:**
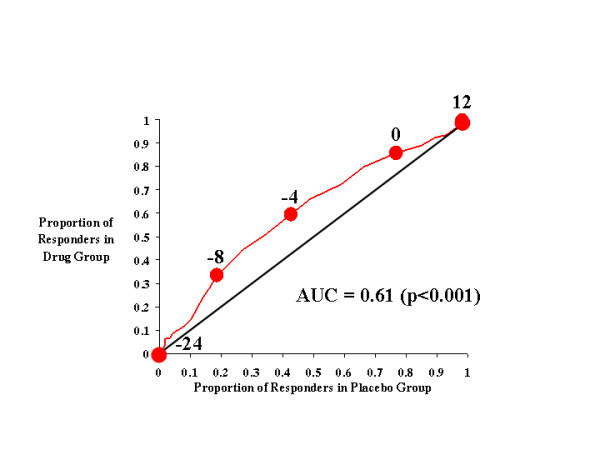
CAARS Investigator-Rated Inattention Subscale.

**Figure 3:**
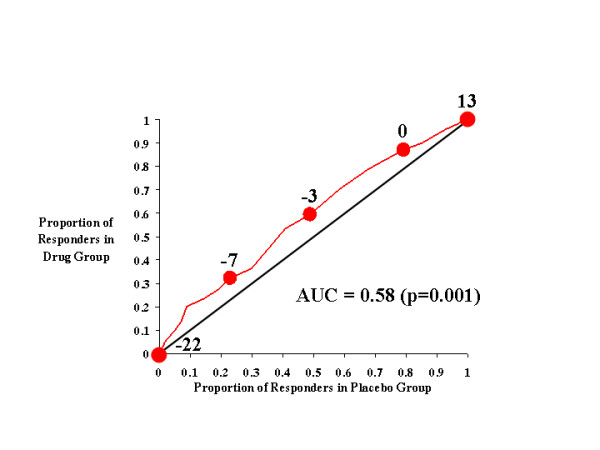
CAARS Investigator-Rated Hyperactive Subscale.

**Figure 4:**
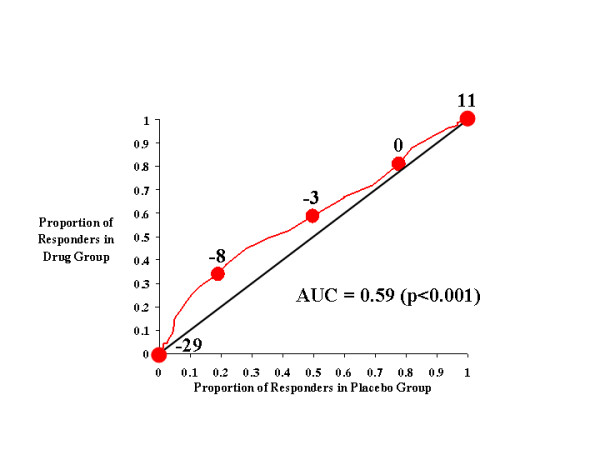
CAARS Investigator-Rated ADHD Index.

Figures 1, 2, 3, 4 show the results for investigator ratings on the CAARS. Figure 1 compares the effects of atomoxetine on total ADHD score with those of placebo. For different definitions of responsiveness (*i.e*., different CAARS cutting scores), the points on the curve illustrate the results of two calculations: the rate of response to drug and the rate of response to placebo. For example, the point in Figure 1 labeled -7 is located at coordinates [0.4, 0.54], which indicates that 40% of those treated with placebo achieved a change of -7 in total investigator-rated ADHD symptoms on the CAARS, whereas 54% of those treated with atomoxetine attained the same level of responsiveness. Sequential points further down the curve (*i.e*., toward the origin) specify increasingly stringent thresholds for defining improvement (*i.e*., larger decreases in investigator-rated total ADHD score), while points further up the curve denote the proportions of each treatment group that responded to treatment as determined by increasingly lenient criteria for improvement. Thus, these points and the curve that joins them illustrate how the drug- and placebo-response rates change as the cutting score used to define improvement is incrementally changed. From the curve, it is clear that if response criteria between total ADHD symptom change scores of -1 and -14 are used as the cutting score, a greater proportion of atomoxetine-treated individuals than placebo-treated patients will attain that level of symptom improvement. In other words, over this range of cutting scores, the majority of individuals judged as responsive to treatment will have received atomoxetine rather than placebo. Near the most extreme cutting scores (in this case, the points labeled 15 and -46), those treated with placebo were as likely as those treated with atomoxetine to reach response criteria.

Figure 1 (and each figure) also shows the diagonal line of no effect, which allows us to visualize the size of the drug effect as the degree to which the drug-placebo response curve rises above it. If outcome on drug were worse than outcome on placebo, then the drug-placebo response curve would fall below the line of no effect; however, as Figure 1 shows, atomoxetine produced a drug-placebo response curve that was always above the diagonal line of no effect. This indicates that atomoxetine outperformed placebo throughout the full range of outcome scores. The area under the curve (AUC) is 0.60, which means that atomoxetine outperformed placebo 60 percent of the time, regardless of cutting score.

In addition to this information, and unlike a traditional statistical analysis, Figure 1 also allows us to determine if the effects of atomoxetine were due to its ability to improve the symptoms of ADHD, prevent their worsening, or both. For example, for CAARS total ADHD symptom change scores, a value of 0 indicates no change, and this point on the drug-placebo response curve is labeled. At this point on the curve, we see that 82% of subjects receiving atomoxetine had a score of 0 or greater, *i.e*., only 18% of atomoxetine-treated patients experienced a worsening of their symptoms. In contrast, 71% of placebo-treated subjects had a score of 0 or greater, which indicates that symptoms worsened in 29% of these patients. Thus, the response curve clearly demonstrates that atomoxetine not only reduced symptoms, but prevented their worsening as well.

Figure 2 illustrates the effects of atomoxetine on investigator-rated inattention on the CAARS. As with total ADHD symptoms, inattentive symptoms seem to be more effectively treated by atomoxetine than placebo, as the drug-placebo response curve was above the diagonal line of no effect and the AUC was 0.61. Relative to total ADHD symptoms, there exists for inattention a narrower range of cutting scores over which the greatest difference in responsiveness was seen between atomoxetine- and placebo-treated subjects. At cutting scores of -4 through -8, approximately 50% more atomoxetine-treated subjects reached the response criterion than did placebo-treated subjects.

Figure 3 shows the effects of atomoxetine and placebo on investigator-rated hyperactivity on the CAARS. Relative to Figure 2 (inattention), the drug-placebo response curve in Figure 3 did not rise as far above the diagonal line of no effect and, consequently, the AUC for hyperactivity (0.58) was lower than that for inattention (0.61). These results indicate that atomoxetine was less effective in reducing (and preventing the worsening of) hyperactivity than in improving attention. However, it is still clear that, relative to placebo, atomoxetine was an effective treatment for hyperactivity when any symptom change score criteria of less than 0 was used as the response criterion.

In Figure 4, the effects of atomoxetine and placebo on investigator-rated ADHD index scores are plotted, and the more restricted rise in this curve is immediately apparent relative to that seen in earlier figures. The AUC for this curve (0.59) was significant (*p *< 0.001), indicating that atomoxetine was more likely than placebo to reduce the ADHD index score. However, there is a distinct peak in this curve at a response criterion of approximately -8, where atomoxetine-treated subjects were approximately twice as likely as placebo-treated subjects to attain this level of symptom improvement; at other cutting scores (*e.g*., -3), the benefits of atomoxetine were much more modest.

### CAARS Self Ratings

**Figure 5:**
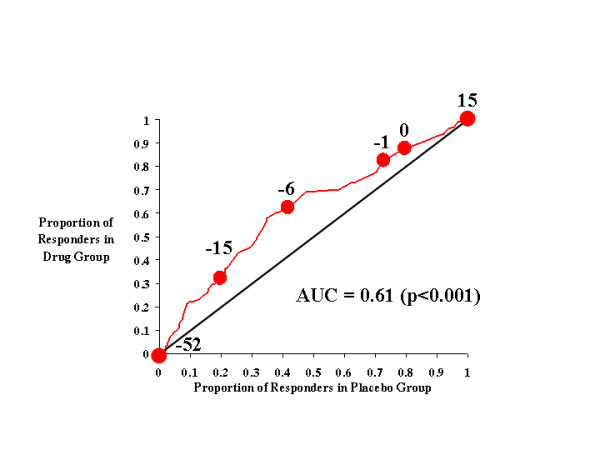
CAARS Self-Rated Total ADHD Score.

**Figure 6:**
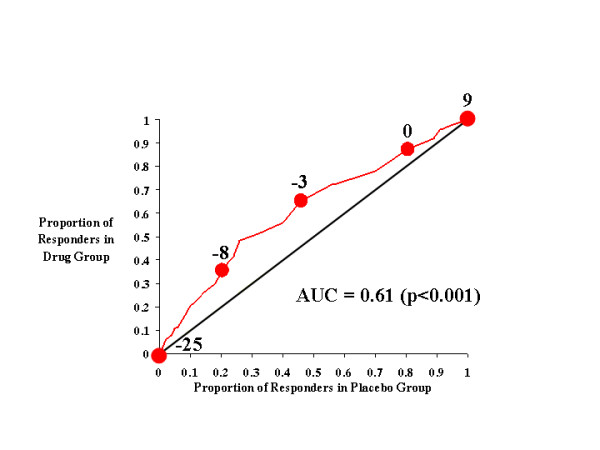
CAARS Self-Rated Inattention Subscale.

**Figure 7:**
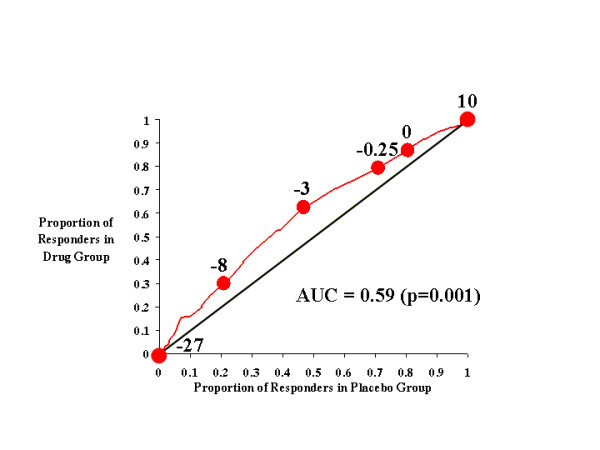
CAARS Self-Rated Hyperactive Subscale.

**Figure 8:**
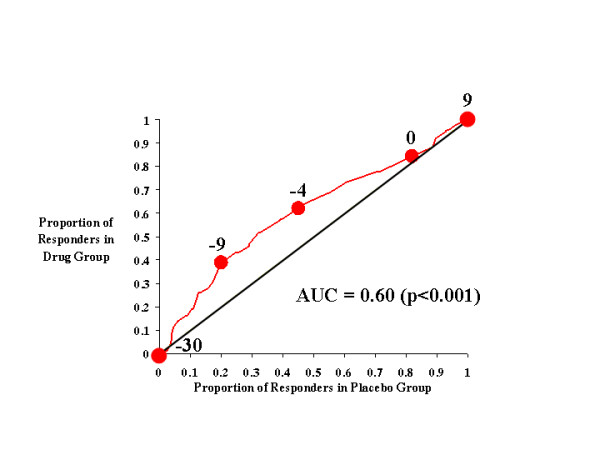
CAARS Self-Rated ADHD Index.

Figures 5, 6, 7, 8, illustrate the results for self-ratings on the CAARS. In general, the effects of atomoxetine on self-ratings on the CAARS mirrored its effects on investigator ratings. From Figure 5 it is clear that, as with investigator-ratings of this measure, self-rated total ADHD symptoms were more likely to be reduced in atomoxetine-treated subjects than in those receiving placebo. In fact, the AUC for this measure (0.61) was virtually identical to that observed on investigator ratings of this measure, and beneficial effects of atomoxetine were observed over roughly the same range of cutting scores. More than 60% of subjects treated with atomoxetine attained the median response score (-6), while only approximately 40% of those treated with placebo saw this level of improvement; thus, when the median response score was used as the cutting score for defining responsiveness, atomoxetine had greater efficacy on self reported total ADHD symptomatology than on investigator ratings of this measure (*cf*, Figure 1).

The shape and position of the drug-placebo response curve for self-rated CAARS inattention change scores (Figure 6) was quite similar to the curve for investigator ratings of this measure (Figure 2). Specifically, the curve was above the diagonal line of no effect and had a significant AUC, indicating that atomoxetine administration was more effective than placebo in reducing inattentive symptoms. Of note, the drug-response curve for self-ratings of hyperactivity very closely matched that for investigator-ratings of this measure (Figure 7), indicating an appreciable amount of divergence in subjects' perceptions of the effects of treatment on their constituent symptom clusters. As expected, self-rated ADHD index change scores mirrored investigator ratings in showing a sizeable benefit of atomoxetine over placebo, especially when symptom change scores in the range of -4 to -9 were used as response criteria (Figure 8).

### CGI Clinician Ratings

**Figure 9:**
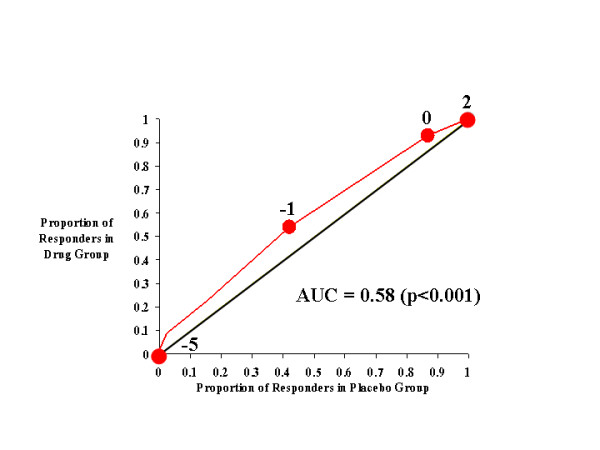
CGI ADHD Severity (Change Scores).

**Figure 10:**
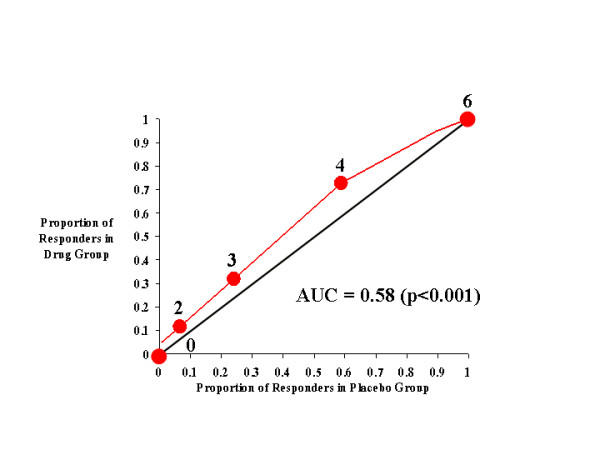
CGI ADHD Severity (Endpoint Scores).

Figure 9 depicts the effects of atomoxetine and placebo on ADHD symptom severity change scores for the CGI. As with the CAARS measures, the drug-placebo response curve occupied the space above the diagonal line of no effect and the AUC approximated 0.60, indicating an advantage of atomoxetine over placebo in reducing clinician-rated ADHD severity. Also in accord with the CAARS measures, a small protection from symptom worsening was afforded by atomoxetine, as approximately 10% of subjects who received the drug deteriorated clinically, while approximately twice as many placebo-treated subjects did so. The median change in CGI in the combined group of atomoxetine- and placebo-treated subjects was -1, a response criterion attained by almost 55% of drug-treated subjects but by only approximately 40% of those receiving placebo. As expected, these greater rates of improvement (and protection from deterioration) led to the attainment of lower CGI endpoint scores in the atomoxetine-treated group (Figure 10).

## Discussion

The results of two large randomized, double-blind, placebo-controlled trials of atomoxetine for the treatment of ADHD in adults were initially reported by Michelson *et al*. [3], who documented the superiority of this compound relative to placebo in reducing total ADHD symptoms, as well as inattentive and hyperactive symptoms of the illness. Due to the ample size and rigorous design of these studies, as well as the strong statistical significance of their results, the efficacy of atomoxetine has been firmly established. However, the simple knowledge that, on average, atomoxetine is efficacious does not tell clinicians much about its full range of effect.

Drug-placebo response curves provide an easily interpretable format for further evaluating clinically informative characteristics of a compound with proven efficacy. Because atomoxetine has demonstrable efficacy, drug-placebo response curve analysis of its performance against placebo was warranted. Collectively, the drug-placebo response curves presented here for each of the different reporters and the various dependent measures paint a consistent picture of the benefits of atomoxetine. First, it is clear that atomoxetine is superior to placebo in reducing total ADHD symptoms as well as individual symptom clusters, such as inattention and hyperactivity. For each of these measures, the drug-placebo response curve was always situated above the line of no effect, indicating that subjects were more likely to respond to atomoxetine than to placebo over the entire range of possible criteria of responsiveness. In addition, it is clear that atomoxetine targeted the core features of ADHD rather than only one of its most conspicuous features of inattention and hyperactivity, as AUCs across total, inattention, and hyperactivity change scores were quite similar. Second, responsiveness to atomoxetine was reliably assessed by clinicians, investigators, and patients, as the AUCs for the various dependent measures varied little (0.58–0.61) across reporters. Third, atomoxetine not only reduced the symptoms of ADHD, but prevented the worsening of these symptoms as well, a finding that has been seen for drug-placebo response curve analyses of other medications [5,10,11]. In contrast however, these prior drug-placebo response curve analyses have also revealed stronger effects of other medications on clinician-rated ADHD symptomatology, as evidenced by AUCs of 0.86 for Adderall [5,10,11], 0.89 for methylphenidate [5,10,11], and 0.93 for desipramine [5,10,11], as compared to the AUC of approximately 0.60 presently observed for atomoxetine.

In conclusion, we have extended the statistical results of Michelson *et al*. [3] by using drug-placebo response curves to describe the clinical significance of the efficacy of atomoxetine in the treatment of ADHD among adults. Our method of data presentation provides readers and clinicians with a means of understanding the nature of the effects of this drug, and the degree to which they are clinically relevant. Rather than collapsing individual responses into means or single rates of response, the drug-placebo response curve illustrates clinically meaningful details that often are lost in a standard analysis, such as the ability of atomoxetine to improve outcome and prevent worsening throughout the full range of outcome scores. The present drug-placebo response analysis provided strong support for the efficacy of atomoxetine relative to placebo for reducing inattention, hyperactivity, and total ADHD symptoms assessed by a variety of reporters, and for preventing the worsening of these symptoms. The finding that atomoxetine is efficacious through the full range of outcome further emphasizes the clinical value of treating ADHD adults with this medication.

## Methods

### Subjects

Two identical randomized, double-blind, placebo-controlled studies were conducted concurrently at 17 (Study I) and 14 (Study II) outpatient sites in North America. Each site's institutional review board evaluated and approved the study protocol, and written informed consent was obtained from each patient. Adults who met DSM-IV criteria for ADHD as assessed by clinical interview and confirmed by the Conners' Adult ADHD Diagnostic Interview for DSM-IV were recruited from clinics and by advertisement. Patients were required to have at least moderate symptom severity, and the diagnosis had to be corroborated by a second reporter for either current symptoms (by a significant other) or childhood symptoms (by a parent or older sibling). Patients who met diagnostic criteria for any other Axis-I disorder using the Structured Clinical Interview for DSM-IV were excluded, as were patients with serious medical illness or habitual substance abuse.

### Atomoxetine and Placebo Administration

Following an initial one-week medication washout and evaluation period, patients entered a two-week placebo lead-in phase. Patients who maintained the initial severity criteria required for study entry were randomized to receive atomoxetine or placebo for a 10-week period. Atomoxetine was administered in evenly divided doses in the morning and late afternoon/early evening beginning at a total daily dose of 60 mg. Patients with residual symptoms received higher doses of up to 90 mg/day after two weeks and 120 mg/day after four weeks. If patients developed problems tolerating this regimen, the dose could be decreased to the last tolerated dose or an increase in dosage could be omitted. Across both studies, 270 subjects received atomoxetine, while 263 subjects received placebo. Of these, 197 completed acute treatment with atomoxetine, while 211 placebo-treated subjects completed the trial, a difference that was not significant.

### Outcome Measures

The outcome measures examined in this study were derived from the CAARS and the CGI. A clinician completed the CGI before and after the treatment regimen, while both the subject and an investigator completed the CAARS before and after treatment. The three groups of primary dependent measures of this study included: 1.) clinician-rated CGI ADHD Severity change scores and endpoint scores; 2.) investigator-rated inattention, hyperactivity/impulsivity, total symptoms, and ADHD index scores on the CAARS; and 3.) self-rated inattention, hyperactivity/impulsivity, total symptoms, and ADHD index scores on the CAARS.

### Drug-Placebo Response Curve Analysis

The rationale and methodology for drug-placebo response curve analysis methods are described in detail by Faraone *et al*. [5] The goal of response curve analysis is not to demonstrate statistically significant group differences; rather, this method provides an alternative means of displaying differences that have already been demonstrated to be statistically significant. Thus, it does not replace a standard statistical analysis, but augments that analysis by showing the clinical significance of drug effects. For the present study, the use of drug-placebo response curve analysis is warranted, as the statistically significant effects of atomoxetine on reducing symptoms of ADHD in adults have been documented previously.

The drug-placebo response curve is constructed in the following six steps: 1.) Choose an outcome variable, for example the change in CAARS Inattention score from baseline to the end of the study; 2.) At each observed score, calculate separately for the drug and placebo groups the proportion of subjects having that score or a better score. For CAARS change scores, therapeutic change is indicated by negative numbers, *i.e*., a decrease in the symptom score; 3.) For each observed score, plot these proportions for the drug group on the vertical axis against the proportions computed for the placebo group on the horizontal axis; 4.) Connect the plotted points and label those that correspond to the best response, the 25^th ^percentile of response, the median response, the 75^th ^percentile of response and the worst response; 5.) If the outcome variable is a change score, also label the point corresponding to no change; 6.) Plot the line of no effect, which is the diagonal line from the [0, 0] point to the [1,1] point. Each point along a curve represents an observed outcome score on that measure, and the points on each plot are then connected by line segments. The line of no effect comprises all points for which the proportion of subjects who respond to drug is the same as the proportion who respond to placebo.

The drug-placebo response curve is a graphical method of describing results from a clinical trial, not a statistical test. It is most sensibly used to describe an effect that has been demonstrated with appropriate statistical tools. Nevertheless, the drug-placebo response curve's roots in (ROC) analysis motivate the computation of one statistic, the AUC, which is computed through integration. The area under the drug-placebo response curve ranges from 0.5 (when the drug effect equals the placebo effect) to 1.0 (when the drug is completely effective and the placebo has no effect). The AUC is a useful index of clinical significance because it equals the probability that a randomly selected member of the drug group will have a better result than a randomly selected member of the placebo group [12,13], *i.e*., the probability that drug will outperform placebo.

In summary, the placebo-response curve provides four pieces of clinically relevant data not typically available from traditional statistical analyses of outcomes data. First, the effect size of a drug on an outcome measure can be determined as the distance between the curve and the line of no effect at any given cut-point. Second, the ratio of drug responders to placebo responders across the range of outcomes can be determined as the area under the curve. Third, the likelihood of a drug to elicit a specific outcome (*e.g*., a clinically meaningful cut-point) can be determined as the proportion of drug-responders to placebo-responders at any given cut-point. Fourth, the ability of a drug to improve functioning *vs*. prevent worsening of functioning can be determined as the proportion of drug-responders to placebo-responders at the outcome score representing no change.

## Competing interests

**Stephen V. Faraone, PhD.- **Stephen Faraone receives research funding from Lilly, McNeil and Shire.

**Joseph Biederman, MD.- ***Joseph Biederman receives research support from the following sources: *Shire Laboratories, Inc and Eli Lilly & Company, Pfizer Pharmaceutical, Cephalon Pharmaceutical,, Janssen Pharaceutical, Neurosearch. Pharmaceuticals, Stanley Medical Institute, Lilly Foundation, Prechter Foundation, NIMH, NICHD and NIDA

*Dr. Joseph Biederman is a speaker for the following speaker's bureaus*: Eli Lilly & Company, Pfizer Pharmaceutical, Novartis Pharmaceutical, Wyeth Ayerst, Shire Laboratories Inc, McNeil Pharmaceutical, and Cephalon Pharmaceutical

*Dr. Joseph Biederman is on the advisory board for the following pharmaceutical companies*: Eli Lilly & Company, CellTech, Shire Laboratories Inc, Novartis Pharmaceutical, Noven Pharmaceutical, McNeil Pharmaceuticals, Janssen, Johnson & Johnson, Pfizer, and Cephalon Pharmaceuticals

**Thomas Spencer, MD- ***Dr. Thomas Spencer receives research support from the following sources: *Shire Laboratories, Inc and Eli Lilly & Company, Glaxo-Smith Kline, Pfizer Pharmaceutical, McNeil Pharmaceutical, Novartis Pharmaceutical, and NIMH

*Dr. Thomas Spencer is a speaker for the following speaker's bureaus*: Glaxo-Smith Kline, Eli Lilly & Company, Novartis Pharmaceutical, Wyeth Ayerst, Shire Laboratories Inc, McNeil Pharmaceutical

Dr. *Thomas Spencer *is on the advisory board for the following pharmaceutical companies: Shire Laboratories, Inc and Eli Lilly & Company, Glaxo-Smith Kline, Pfizer Pharmaceutical, McNeil Pharmaceutical, and Novartis Pharmaceutical

**David Michelson, MD- **David Michelson is a Lilly employee

**Lenard Adler, MD- **Lenard Adler receives grant and Research Support, is a Consultant or on Advisory Boards: Abbott Laboratories, Bristol-Myers Squibb, Eli Lilly and Co., McNeil/Johnson & Johnson, Merck & Co., Inc., Neurosearch, Novartis Pharmaceuticals Corp., Pfizer Labs, Cortex Pharmaceuticals, Cephalon and Shire Pharmaceuticals

**Fred Reimherr, MD-**Fred Reimherr has been part of Lilly advisory board.

**Stephen J Glatt, PhD- **Stephen Glatt has no conflicts of interest to declare.

## Authors' contributions

**Stephen V. Faraone, PhD- **Steve Faraone contributed to the analysis and interpretation of the data, the drafting and revision of the manuscript.

**Joseph Biederman, MD- **Joseph Biederman contributed to the analysis and interpretation of the data, the drafting and revision of the manuscript.

**Thomas Spencer, MD- **Thomas Spencer contributed to the conception and design of the study, the acquisition of data, interpretation of data and drafting/reviewing the manuscript.

**Lenard Adler, MD- **Lenard Adler contributed to the conception and design of the study, the acquisition of data, interpretation of data and drafting/reviewing the manuscript.

**David Michelson, MD- **David Michelson contributed to the study conception design, the data acquisition as well as critical reviewing of the manuscript.

**Fred Reimherr, MD- **Fred Reimherr contributed to the study conception design, the data acquisition as well as critical reviewing of the manuscript.

**Stephen J Glatt, PhD- **Stephen Glatt contributed to the analysis and interpretation of the data, and the drafting and revision of the manuscript.
